# Youth Justice Services: Relationships, Rehabilitation and the Reality of the Young People Involved—A Meta-synthesis of the Qualitative Literature

**DOI:** 10.1007/s10567-025-00534-6

**Published:** 2025-07-09

**Authors:** Kerri Moore, Elayne Ahern, Eoin O’Meara Daly, Sharon Houghton, Hannah McAuliffe, Elaine Rogers

**Affiliations:** 1 Department of Psychology, Faculty of Education and Health Sciences, University of Limerick, Limerick, V94 T9PX Ireland; 2 REPPP Project, School of Law, University of Limerick, Limerick, V94 T9PX Ireland; 3 University Hospital Limerick, Co., Limerick, V94 F858 Ireland

**Keywords:** Youth justice, Young people, Professional relationships, Diversion, Restorative justice, Qualitative evidence synthesis

## Abstract

Rehabilitative approaches to working with young people in the youth justice system have been identified as effective in reducing recidivism, with the centrality of relationships being core to these approaches. There is a limited evidence base exploring young people’s experience of professional relationships within youth justice systems. This review aims to explore young peoples’ experience of relationships with professionals within youth justice services and understand what is important to their engagement. Five databases were systematically searched for relevant peer-reviewed qualitative and mixed-method journal articles. Seven papers were included in this review. Data were analysed using a meta-synthesis approach. PRISMA and ENTREQ guidelines were followed. Analyses yielded four themes: Feeling valued and finding worth in the system; The reciprocal nature of understanding and respect; nobody trying to help and no one to try for; and the importance of having one good person. The importance of the relationships between youth justice professionals and young people are discussed. Limitations and implications for research, practice and policy are also discussed.

## Introduction

In recent years, there has been a reduction of recorded youth crime internationally. These trends have been documented in both official police documents and through independent studies (Svensson & Oberwittler, 2021; Baumer, 2018). These declining trends have been highlighted in several countries, including the United States (Arnett, 2014), New Zealand (Polglase & Lambie, 2024) and across Europe (Fernández-Molina & Bartolomé Gutiérrez, 2020; Griffiths & Norris, 2020; van der Laan et al., 2021; Vasiljevic et al., 2020). In Ireland, youth crime has significantly reduced, with reported incidents decreasing by a third from 2007 to 2019 (An Garda Síochána, 2019). Similar findings have been reported across youth justice services in the United Kingdom (HMIP, 2021).

The notable decrease in recorded youth crime has led many policy makers, professionals, and researchers to ask the question; what is working to effect this change? There remains a thin evidence base for evaluating programmes developed for addressing ‘juvenile delinquency’, presenting a challenge to practitioners and policy makers wanting to introduce evidence-based approaches (Wilson & Lipsey, 2024). There have been significant changes and reforms within the juvenile justice system, moving away from retributive models and towards rehabilitative models focussed on young people’s development (April et al., 2023). Comparisons across the literature have found therapeutic approaches to be more effective than behavioural control approaches in reducing recidivism. For example, findings reported as part of a systematic review (Evans-Chase & Zhou, 2014), whereby a narrative synthesis of quantitative outcomes from intervention studies, utilizing a control group, found that therapeutic approaches, as compared to behavioural control approaches, were more effective in reducing recidivism. The authors of this review found only 21 studies, out of a potential 141 spanning 13 years, met the quality inclusion criteria, indicating a lack of rigour in study design. They named treatment fidelity as the cause of quality inclusion failure. Where fidelity to treatment was adhered to in the studies, 88% reported an improvement in recidivism rates when compared to control groups. Whilst 120 studies were excluded from the systematic review, this speaks to study design and publication practises rather than a lack of evidence in support of therapeutic approaches to reducing recidivism (Evans-Chase & Zhou, 2014).

Additionally, a meta-review on what works for young people in the youth justice system found that interventions focussed on young people’s needs relating to multisystem and family based treatment, with a combination of rehabilitative and deterrence related approaches, had the most powerful impact on recidivism. However, due to the limitations common to meta-reviews, in that the reported effect sizes are averages of averages, the true effect size is difficult to ascertain. Additionally, causal inferences could not be made as some studies did not discriminate between experimental and non-experimental designs. Given the volume of studies which support a combination approach to offending interventions, and notwithstanding that the effect size may be less clear, it can be said with relative certainty that such programmes are more effective at reducing recidivism (Pappas & Dent, 2023).

There are currently three main competing rehabilitative approaches to juvenile justice reform: the public health model, the social-ecological model, and the restorative justice model (April et al., 2023). Core to each of these approaches is the centrality of relationships: youth crime interventions should be relational, long-term and supportive (Creaney, 2014). Arguably, relying on recidivism as the primary success metric within youth justice interventions fails those impacted by trauma, potentially lengthening their justice involvement. A holistic approach incorporating relational health and wellbeing should therefore be used to measure success (Zelechoski et al., 2024). The importance of trusted relationships within children’s services is well documented within the literature (Lewing et al., 2018). Practitioners have recognised the importance of relationships between young people and themselves, reflecting that the “nuances, boundaries and detailed dynamics” involved in the relationship may affect change (Drake et al., 2014). However, despite the centrality of relationships across the dominant models, there is limited evidence on the importance of relationships within youth justice services (Fullerton et al., 2021), and of the efforts to understand the role of relationships within these settings, research has primarily focussed on the perspectives of professionals (Lohmeyer, 2020).

A collaborative therapeutic alliance is suggested to be pivotal for preventing recidivism and promoting positive youth development (Ayotte et al., 2016). A collaborative relationship between therapist and client has been coined a therapeutic alliance within psychotherapy literature (Bordin, 1979). Similar terminology has been documented within youth justice literature, with research focusing specifically on the role of the alliance between youth justice professionals and young people involved in the justice system (Brown et al., 2014). Treatment benefits are likely associated with the strengthening of the alliance when treating justice-involved youth, with an established positive alliance setting the foundation for initiating change (Papalia et al., 2022). Relational working within youth diversion projects is central and critical to success, in that if there is no relationship between youth justice workers and young people, then there will be no intervention (Redmond, 2009).

The UN Committee on the Rights of the Child emphasises the embracement of children’s voices and underpins children’s rights within the justice system (United Nations, 2019). This was operationalised in a model which further supports this move, insisting that children must be given safe, inclusive opportunities to form and express their view, that children must be supported to express their view, that their view must be listened to by others and their view should be acted upon appropriately (Lundy, 2018; Welty & Lundy, 2013). Within youth services, shared decision-making, involving an equal partnership of a collaborative nature, can transform relationships between young people and professionals (Martin & Feltham, 2020; Redmond et al., 2015).

However, there remains a limited body of evidence on the impact of relationships within youth justice services from the perspectives of the young people involved. In addressing this gap, this systematic review aims to synthesise available qualitative data that focuses on young people’s experience of relationships with professionals within youth justice services. The research questions specifically address (i) what are young people’s views and experiences of the relationships they have with youth justice professionals? and (ii) what factors have been identified by young people as important to their engagement with professionals?

## Materials/Methods

The protocol for this systematic review was pre-registered with the Open Science Framework. This review was guided by the Preferred Reporting Items for Systematic Reviews and Meta-Analysis (PRISMA; Page et al., 2021), and Enhancing Transparency in Reporting the synthesis of Qualitative research (ENTREQ; Tong et al., 2012). The review was informed by Lachal and colleagues (2017) method on synthesising qualitative literature. This method follows six steps: define the research question and the inclusion criteria, select the studies, assess quality, extract/present formal data, analyse data and express the synthesis (Lachal et al., 2017). The quality of the included research studies was evaluated using the Critical Appraisal Skills Programme (CASP) guidelines (Critical Appraisal Skills Programme, 2018).

### Search Strategy

Systematic searches were undertaken using five databases: PsycInfo, Medline, Embase, Web of Science, and National Criminal Justice Reference Service Abstracts. The reference lists of included papers were also searched for papers that met the inclusion criteria for this study. The searches were completed on 13th May 2023.

For the purpose of the search strategy, search terms included various labels and descriptions for young people involved in youth justice services. This approach was adopted in order to capture all relevant literature, particularly research conducted across different timelines, where guidance on specific language had not yet been modernised to reduce the stigmatisation of marginalised groups (Jordan, 2017). However, despite being used in the search strategy to source relevant evidence, labels such as “offenders” or “delinquents” will not be used in this paper. Ongoing use of stigmatising labels is an ethical issue. It is a practice that may lead to obstruction of the desistance process, to young people feeling ostracised, and potentially broadening the gap between youth justice-involved young people and conventional society. For those involved in the justice system to feel part of society, they must be accepted as the individuals they are (Willis, 2018). Participants in the included studies and those discussed within the literature will be referred to as ‘young people’ in the present review, to preserve their unique identities, and to not categorise or stigmatise them further.

### Inclusion/Exclusion Criteria

Peer-reviewed, full-text, qualitative studies describing young people’s experiences of relationships with staff within justice related services were included. No age limit was applied to the sample, as age specifications across countries, legal systems, and services vary. The minimum age of criminal responsibility varies internationally. A modern method considers if a young person can contend to the moral and psychological components of responsibility, that is, whether a young person, on account of their judgement and understanding, can be held responsible for their behaviour (United Nations, 1985). The sample was therefore defined by the services in which the young people were engaged in, i.e. juvenile justice related services specifically co-ordinated by a local authority, to capture young people’s experience of being involved in professional relationships that were not of their initial designation. Mixed methods studies were included where qualitative findings were reported in order to avoid missing relevant data.

The following studies were excluded from the meta-synthesis: (1) Studies in languages other than English; (2) Commentary, reports that were not peer-reviewed, case studies, dissertations or reviews; (3) Studies describing young people’s experiences of relationships with professionals separate to the core service or team, i.e. relationships with professionals independent from the core team, professionals delivering programmes within the service that had the option for voluntary engagement; or relationships with professionals from voluntary sector services, separate to statutory youth justice services; (4) Retrospective accounts, i.e. whereby the young person was no longer involved in youth justice services and was reflecting back on their experiences, or a young person in adult justice services reflecting or comparing their time in youth justice services; (5) Studies deemed not to contain relevant data. That is, studies were excluded if a different relationship or outcome was being explored. Studies were also excluded when less than 50% of the qualitative analysis focussed on the relevant relationship between the young person and staff within the justice service.

### Data Screening and Extraction

**Fig. 1:**
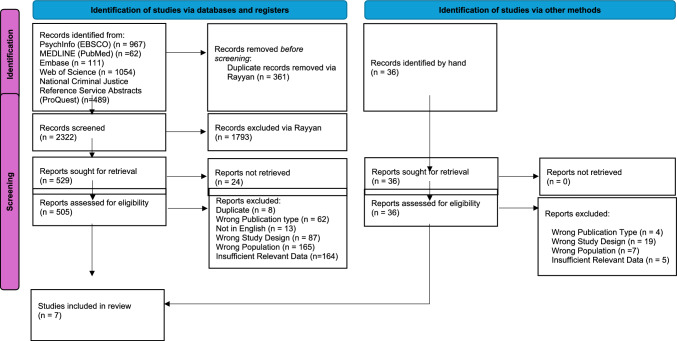
PRISMA flow diagram of identified studies

Screening and study selection were conducted in accordance with PRISMA guidelines (see Fig. 1). Title and abstracts were screened using Rayyan software, by two researchers. Any discrepancies between Reviewer 1 and Reviewer 2 were discussed and resolved. Reviewer 1 screened all full-text studies. Reviewer 2 screened 20% of all full-text studies. EndNote 20 was utilised for the management of references and Rayyan software was used for the recording of decisions. Data were extracted by Reviewer 1 and included title, authors, year of publication, country, study design, research aims, sample size, participant characteristics (age range, gender, service, staff role) and findings, which were entered into an Excel spreadsheet.

### Quality Assessment

The quality of included studies in the meta-synthesis was assessed using the CASP qualitative research checklist, to support review robustness and content assessment (Critical Appraisal Skills Programme, 2018). The quality appraisal was carried out in full by Reviewer 1 and 10% by Reviewer 2. Any discrepancies were discussed with all authors of this review. All studies were deemed acceptable for inclusion. Table 1. shows a summary table of the results from the quality assessment completed on the included papers using CASP guidelines (Critical Appraisal Skills Programme, 2018). As per CASP recommendations, a scoring system was not used but rather an assessment of the strengths and weaknesses of the papers included in reference to answering the research question and aims of the current review (Tong et al., 2012).

**Table 1:** CASP quality assessment of included studies

	1. Was there a clear statement of the aims of the research?	2. Is a qualitative methodology appropriate?	3. Was the research design appropriate to address the aims of the research?	4. Was the recruitment strategy appropriate to the aims of the research?	5. Were the data collected in a way that addressed the research issue?	6. Has the relationship between researcher and participants been adequately considered?	7. Have ethical issues been taken into consideration?	8. Was the data analysis sufficiently rigorous?	9. Is there a clear statement of findings?	10. How valuable is the research?
Brubaker and Cleary (2023)	T	T	T	T	T	T	T	T	T	T
Cross (2020)	T	T	T	T	T	T	T	T	T	T
Jacob et al. (2024)	T	T	C	T	T	T	T	T	T	T
King (2022)	T	T	T	T	T	N	T	T	T	T
Phoenix and Kelly (2013)	T	T	C	C	N	N	C	C	T	T
Tarrant and Torn (2021)	T	T	T	T	T	T	T	T	T	T
Trivasse (2017)	T	T	T	T	T	T	T	T	T	T

T, totally met, C, can’t tell, N, not met

The findings of this quality review process indicated that four studies (Brubaker & Cleary, 2023; Cross, 2020; Tarrant & Torn, 2021; Trivasse, 2017) were deemed to be of high quality, satisfying all 10 items on the CASP. In one study (Jacob et al., 2024) it was not possible to tell if the research design was appropriate to address the research aims. Two other studies might be deemed of lower quality because of the reporting of the researcher-participant relationship (King, 2022; Phoenix & Kelly, 2013) or the methodological choices for data collection, or reporting of same (Phoenix & Kelly, 2013). Collectively, the quality of the body of evidence was determined to lend itself to reliable data.

### Meta-synthesis Procedures

The meta-synthesis was informed by Lachal and colleagues (2017) method on synthesising qualitative literature. The authors extracted both first order data, which included direct quotations, and second order data, which included previous authors interpretations. This was entered into an Excel spreadsheet. Coding was completed line by line using an inductive approach, which involves reading and interpreting raw textual data to construct concepts and themes through interpretations based on the data (Chandra & Shang, 2019), whilst attempting to stay as close as possible to the participant’s own words (Lachal et al., 2017). Codes were grouped and categorised into a hierarchical structure. In the final stage of the analysis, codes were discussed with all authors across multiple meetings and analytical inductive themes were developed by the team with reference to the research questions: (i) what are young people’s views and experiences of the relationships they have with youth justice professionals? and (ii) what factors have been identified by young people as important to their engagement with professionals?

### Reflexivity

As discussed by Lachal and colleagues (2017), the data analysis step is the most subjective of the steps and the most influenced by the authors’ backgrounds. It is paramount to assess rigour and it is suggested that triangulation is enhanced when researchers with different backgrounds are involved in the analysis of the same data set (Cho & Lee, 2014). ER is an academic clinical psychologist at the University of Limerick, EA is an Associate Professor and Psychology Co-ordinator at the University of Limerick, EOD is a Senior Research Fellow in Youth Justice and Module Leader in Youth Crime at the University of Limerick and KM is a doctoral Clinical Psychologist in training. In attempts to promote awareness and mitigation of bias, the team engaged in critical reflective discussions throughout the research process.

## Results

### Study Selection and Characteristics

This review captures the views of 150 young people. Five of the papers used qualitative methodology exclusively, one paper followed a mixed-method approach of both qualitative and quantitative methodology, and one paper used secondary data analysis of a data subset, which used a case study methodology. Of the studies that reported age and gender demographics, the age range of participants was 14–20 years old, 16% were female and 84% male. Four of the studies were conducted within Youth Offending Teams (YOTs), with the remaining three studies being conducted in settings under the umbrella of correctional institutions. The majority of studies originated in the United Kingdom (*N* = 6) and one study originated in the United States. Full description of the studies can be seen in Table 2.

**Table 2:** Study characteristics

Study no.	Researchers and country	Participant characteristics and sample size	Setting/involvement	Staff’s role	Study design	Data analysis	Study aims	Findings
1	Brubaker and Cleary (2023) Virginia, United States	Aged 15–20, Gender unknown *N* = 69	State-run juvenile correctional facility	Resident and security specialists	Mixed-method exploratory inductive study using focus groups and quantitative survey	Thematic, open coding approach	To examine resident and staff member perceptions of a newly implemented therapeutic model in a large (284-bed) state-run juvenile correctional facility. The goal was to identify major themes that emerged from focus groups related to engagement and safety, as well as general perceptions of the model, to increase understanding of the experiences of residents and staff members	*Main qualitative findings*: The Dual Role of Juvenile Justice Staff Structural Components: Staff-resident ratios, Consistency in unit staff, Mentors/personal advocates, Conflict resolution Interpersonal Dimensions: Staff value relationships and connecting with youth, Residents feel that staff care
2	Cross (2020) Wales, United Kingdom	Aged 14–17, males *N* = 5	Youth offending teams	Youth offending team practitioners	Qualitative research framework using semi-structured interviews and focus groups	Thematic analysis	To explore the characteristics of a ‘good quality’ working relationship from two different perspectives: (1) the practitioners and (2) the children	*Main themes*: Trust and Genuineness Reliability Time Comfort
3	Jacob et al. (2024) England, United Kingdom	Aged 16 + , Gender unknown *N* = 28	Secure children’s homes, training centres and young offenders institutes	Specific staff roles not defined	Qualitative research using semi-structured interviews and focus groups	Framework method and grounded theory	To fill a research gap, exploring child-staff therapeutic relationships in the CYPSE in England, by answering the research question: ‘what are the key elements of the development and maintenance of effective therapeutic relationships in the CYPSE?’ ‘Effective’ in this context relates to the impact on children’s experience of the CYPSE, their sense of self and future outlook, including mental health, wellbeing and likelihood of reoffending	*Main themes*: Helping behaviour Instability Lack of facilitative relationships Building facilitative relationships Sense that staff cannot help Communication Understand children and/or express empathy Caring Good Staff Trust Reciprocal respect Treat them like criminals Sense of fair treatment
4	King (2022) England, United Kingdom	Aged under 18, Gender unknown *N* = 5	Youth offending team	Youth offending team workers	Free associative method and the grid elaboration method	Thematic analysis and psychosocial analysis	To influence educational psychology practice by sharing key themes from research and work capturing young peoples’ experiences of engaging with youth offending services (YOS)	*Main themes for thematic analysis*: Transformative relationship with YOT worker Identity transformation Engaging Then’s presence in now *Broad themes from thematic analysis and broader literature*: The importance of trust in relationships; past and present The importance of developing an identity distanced from a past self Engagement in personalised intervention The function and structure of YOS YOS engagement; a window of opportunity Developing a psycho-social understanding of what participants talked about
5	Phoenix and Kelly (2013) England, United Kingdom	Aged 14–17, 25 males, 4 females *N* = 29	Youth offending team	Youth offending team practitioners	Secondary data analysis of an original study which used case study methodology	Thematic ally analysed	To analyse and investigate the social, political and ideological conditions in which youth justice practitioners, lay magistrates, police and solicitors assessed the risks and needs of and made decisions or recommendations about young lawbreakers; and to describe and analyse the engagement (or not) of young people in those processes	*Main themes*: Young people’s sociological accounts of offending It’s the relationship that counts No one cares No one listens, no one understands Going to youth offending does not help You have to do it yourself
6	Tarrant and Torn (2021) England, United Kingdom	Aged 15–18, males *N* = 3	Young offenders institute	Prison officers	Qualitative research using semi-structured interviews	Inductive thematic analysis	To explore the ways in which young people and prison staff (Prison Officers) within a youth custodial establishment experience empathy, and the role of the custodial context in shaping empathy, including the potential impact of relationships, environmental factors and culture	*Constructed themes*: Constructions of empathy Recipe for empathy Institutional Investment The value of empathy Doing empathy
7	Trivasse (2017) England, United Kingdom	Aged 14–18, 9 males, 2 females *N* = 11	Youth justice service	Young offending team practitioners	Qualitative service evaluation using semi-structured interviews	Thematic analysis	To fulfil national guidelines for greater participation of young people within Children’s Services and specifically explored young offenders’ (YOs) views and experiences of the Youth Justice Service (YJS)	*Main themes*: The journey through WYJS Relationships with the YOT

### Findings of the Meta-synthesis

The thematic meta-synthesis generated four themes and two sub-themes; Theme 1—*Feeling valued and finding worth in the system,* Theme 2*—The reciprocal nature of understanding and respect,* Theme 3*—Nobody trying to help and no one to try for,* Theme 4*—The importance of having one good person,* with Sub-theme 1—*Creating a secure base for exploration and development,* and Sub-theme 2*—Showing genuine care by going above and beyond. Themes and sub-themes, including study endorsements can be seen in* Table 3.

**Table 3:** Study Endorsements of Themes and Sub-Themes

Themes and sub-themes	Brubaker and Cleary (2023)	Jacob et al. (2024)	King (2022)	Tarrant and Torn (2021)	Cross (2020)	Trivasse (2017)	Phoenix and Kelly (2013)
Theme 1: Feeling valued and finding worth in the system	✓	✓	✓	✓	✓	✓	✓
Theme 2: The reciprocal nature of understanding and respect	✓	✓		✓	✓	✓	✓
Theme 3: Nobody trying to help and no one to try for	✓	✓	✓	✓		✓	✓
Theme 4: The importance of having one good person	✓	✓	✓	✓	✓	✓	✓
Sub-theme 1: Creating a secure base for exploration and development	✓	✓	✓	✓	✓	✓	✓
Sub-theme 2: Showing genuine care by going above and beyond	✓	✓	✓	✓	✓	✓	✓

#### Theme 1: Feeling Valued and Finding Worth in the System

Theme 1 reflects how supportive relationships with youth justice professionals facilitated the development of worth and value in both the young people themselves and also in their engagement in the youth justice system.

Young people reported at first being pessimistic about entering services. Their past experiences impacted their ability to trust, and they were initially cautious of professionals.

"…they had learnt over time that it was safer for them not to trust, including practitioners. It was mentioned frequently by the children, that they were able to spot practitioner insincerity very quickly." (Author quote; Cross, 2020).

Some young people reported being surprised by the treatment they received from professionals and amazed that people cared about them.

"It made me feel a bit surprised and happy as well…That there’s staff here that care about me, like they, there’s random people that care about you as well." (Participant quote; Tarrant & Torn, 2021).

Young people felt valued when professionals focussed less on why they were in the system and cared more about their future, seeing them as a person with potential.

"…they don’t care what you’re in here for, they care about what you’re doing now to improve your life when you get out." (Participant quote; Jacob et al., 2024).

Young people felt recognised as a person when workers took the time to listen, attempting to understand what happened to them and getting to know them better.

"…it was that formulation meeting […] it tells them a bit more about me […] so I’m not just some criminal, I’m actually a person to them now." (Participant quote; Jacob et al., 2024).

When professionals chose to understand young people’s behaviour instead of reacting punitively, it reflected a collaborative approach to conflict resolution, where the young person and their experience were valued.

"…emphasis on conflict resolution helped to humanize each group and contributed to interpersonal shifts in the form of stronger relationships, greater rapport and trust, and a stronger sense of empathy between residents and staff." (Author quote; Brubaker & Cleary, 2023)

Through this supportive relationship, young people described developing a sense of worth in the system, feeling listened to and not judged by professionals.

"…supportive nature offered a sense of relief and allowed individuals to find worth in the system." (Author quote; Trivasse, 2017).

"…helpful having someone there that I can sort of, explain things to, who will actually listen and not sort of, judge me." (Participant quote; King, 2022).

Feeling comfortable with the worker was also linked to the development of self-worth, whereby a worker getting to know them and making them feel comfortable, was another way in which the young person felt valued.

"It might be that the characteristic of feeling comfortable around the practitioner is tied closely to a child's self-worth […] When children get the sense that practitioners genuinely want to understand them and to help them, this serves to increase the child’s self-esteem." (Author quote; Cross, 2020).

One young person described feeling valued and understood in their individual interventions, reporting that the professional took time to adapt the work to suit their ability and go at their pace. This reflects how the worker held the young person in mind and put their needs first.

"…and the individualised interventions, [YOT worker] does sessions but she does, em, sort of at a pace, instead of just going through everything you’re supposed to do." (Participant quote; King, 2022).

This was supportive of their engagement in the service, through feeling they mattered to someone else, they began to matter to themselves. In being valued and treated as a person, some young people discussed starting to value their emotions and feeling comfortable to show their real self.

"I’m not just erm, putting on a show. I can actually, I feel like I can talk to somebody because I kind of know that I will feel those things that I’m saying." (Participant quote; Tarrant & Torn, 2021).

In understanding the young person, the worker modelled empathy, facilitating the young person’s engagement in empathy, which was supportive in developing a more positive sense of self.

"Both giving and receiving empathy also challenged participants’ view of themselves, giving them a sense of themselves as caring, and providing a way of achieving retribution for past wrongdoing." (Author quote; Tarrant & Torn, 2021).

#### Theme 2: The Reciprocal Nature of Understanding and Respect

This theme reflects how professionals’ demonstration of mutual understanding and respect in their interactions with the young people, facilitated the young people to feel more willing to engage and reciprocate these dynamics and form stronger connections with both the professionals and the service.

Both mutuality and reciprocity played an important role in the relationships between young people and professionals. Young people described showing professionals respect, good behaviour and engagement, in the hope that staff would reciprocate this.

"It’s all about how you approach them […] as long as you give them respect, they’ll give it to you." (Participant quote; Jacob et al., 2024).

Some young people reflected that receiving respect was contingent on their behaviour, which may speak to the unspoken power imbalance within the relationship dynamic.

"At times this was explained as being contingent on the behaviour of the child and mutual respect." (Author quote; Jacob et al., 2024).

However, some young people were able to identify and accept the difference in power, on the basis of there being mutual respect and understanding within the relationship.

"…like they hold the power above you, yeah you’re supposed to know that anyway, um, but if you show them respect they’ll show you respect back." (Participant quote; Trivasse, 2017).

For some young people, there was a need to reciprocate care based on a felt sense of responsibility to the worker. Perhaps reflecting the respect they had for the worker and the value they held for their care, it was worth something so great that it had to be paid back.

"I think I’m duty bound to care for them and look out for them because they help me, so I’d feel wrong, I don’t think it’d be equal if I didn’t look out for them." (Participant quote; Tarrant & Torn, 2021).

Young people reported appreciating professionals taking the time to explain the reasons for their actions or why certain procedures were in place. This was a subtle way of showing respect and building a mutual understanding between them.

"When they understand how you feel and they compromise with you. They’ll pull you to the side and let you know. I feel like if you can’t do it, and he points this out like, 'Look. I can’t do this, and this is the reason why…' Even if they say 'because it’s my job on the line,' I feel like they still care because they understand how you feel." (Participant quote; Brubaker & Cleary, 2023).

A mutual understanding also facilitated young people in reflecting and making sense of professionals’ behaviours and empathising with their experiences.

"…if they’re having a bad day it could affect us as well a bit, so we just need to like understand them a bit more and put us in their shoes, like the job they’re doing could be harder sometimes, stressful." (Participant quote; Tarrant & Torn, 2021).

Sharing a mutual understanding with professionals supported young people in connecting with them. It also facilitated young people in connecting and identifying with workers, which opened communication channels between them.

"…me and him have always been on good terms, understand each other and where we’re coming from." (Participant quote; Jacob et al., 2024).

"I find it easier to speak to people who are more similar to me and have the same interests and things like that." (Participant quote; Tarrant & Torn, 2021).

#### Theme 3: Nobody Trying to Help and No One to Try For

Theme 3 highlights differences in experiences for many of the young people, reflecting how relationships can have a paradoxical effect, in comparison to Theme 1. Incapsulated in this theme is how professional relationships can also lead to young people to experience feelings of inadequacy, unimportance, and worthlessness, resulting in them losing hope in the system and creating barriers for meaningful engagement.

Some young people reported never having anyone in their life who they could rely on and discussed how this absence meant there was never anyone to answer to.

"Nick said why did I offend. I don’t know, I said, because there’s no one there to stop me, if you know what I mean. There weren’t no one there to stop me. I was just like on my own." (Participant quote; Phoenix & Kelly, 2013).

For some, they had such a long history of being let down that hope was difficult to muster, leading to a sense of hopelessness, a lack of desire to seek help and a fixed narrative that doing so would be pointless.

"Further, a sense of helplessness was also discussed, where, despite actors in the system being willing to help, the experiences of participants had not changed." (Author quote; Jacob et al., 2024).

"How can they help me? I’m in the office five minutes. How can they get me money or a house? They can’t help me." (Participant quote; Phoenix & Kelly, 2013),

For some young people, when workers provided support, it felt like a limited supply that was variable and easily exhausted.

"Sometimes I feel like I’ve exhausted officers through different things that I’ve done previously and they’re very reluctant to help me. I think that would be a good way to say. Or very reluctant to listen. They may understand but they don’t want to do anything about it or listen or try and follow things up." (Participant quote; Tarrant & Torn, 2021).

Other young people described that when engaging with the worker, the worker seemed to have their own agenda and was not really focusing on them.

"They write whatever they think down. Like I say this is what’s going on in my life and they may or may not write it down. They write what they want to see and what fits their forms. (Participant quote; Phoenix & Kelly, 2013).

And when workers used formal techniques, young people felt this was insincere or that they were just ticking a box. With some adopting a similar attitude, also just ticking a box, rather than engaging meaningfully.

"Using formal techniques was a hindrance in forming a relationship with his YOT practitioner because it felt insincere and generic." (Author quote; Trivasse, 2017).

A similar narrative was echoed in the data, with some feeling workers were just doing what they were paid to do, without holding any regard or respect for the young person.

"…just give you the silent treatment… They be like, 'I’m just here to sit here and watch you, not to think about you.'…That’s the vibe I get from some staff." (Participant quote; Brubaker & Cleary, 2023).

For others, this lack of regard and disrespect went further, and they described ways in which they felt judged, labelled and generalised within the system, expressing a sense of being de-humanised.

"They don’t know your story […] they just see you as that character, [we are] all seen as prisoners, all in the same category." (Participant quote; Jacob et al., 2024).

Whilst other young people reported ways in which workers made their feelings towards them known by passing negative remarks about the young person, sharing their personal information with others or making light of their struggles. One young person reflected on a comment made by a worker that hurt them and confirmed what they already felt.

"Yeah, and when they don’t care is when they always slipping jabs like, 'I ain’t the one locked up, I can go home the next day, I can go home at this time. You gonna be here, not me.' That hurts. And that’s when I know they don’t care." (Participant quote; Brubaker & Cleary, 2023).

#### Theme 4: The Importance of Having One Good Person

Theme 4 identifies how the relationship with professionals provided young people with at the very least one good person in their life, a person who created a secure base for them by showing genuine care and going above what was expected, supportive of their ability to explore and develop within themselves.

For some of the young people who formed a positive relationship with professionals, it was their first experience of trusting someone, as one young person discussed.

"I don’t like trusting people much and (YOT worker) pretty much, well the first person I trusted apart from my best mate… first person I trusted really." (Participant quote; King, 2022).

For some young people, this was their main source of support and who they went to when they needed something, demonstrating the significance of this relationship and the influence the worker could potentially have on them.

"…building up that trusting relationship. To the point where […] the first people they want to talk to is the staff because of the relationship they have […]. If there’s no trust, then the young people can’t get any help for any of their behaviours." (Participant quote; Jacob et al., 2024).

For others who had family or other supports in their lives, the worker was that one person they could bring anything to, with the young people knowing they could tolerate their distress.

"The meetings with [YOT practitioner], they’ve been especially helpful cause like if I don’t know who to talk to like […] my Mum and Dad were going through a lot, so I didn’t want to talk to them about it cause I didn’t wanna put more stress on them." (Participant quote; Trivasse, 2017).

With the powerfulness of the relationship, there was equal fragility, owing to how important and solitary it was for some young people. This perhaps highlights the immense responsibility that comes with maintaining these high expectations and the pressure of getting it right, potentially unbeknownst to some workers.

"For many children trust does not come easily because they have either never had a trusting relationship before, or they had once trusted but had been let down." (Author quote; Cross, 2020).

#### Sub-theme 1: Creating a Secure Base for Exploration and Development

Trust played a central role in both the development and maintenance of the relationship between the young person and the worker. It also formed the foundations for which the young people could engage and benefit from the interventions within services.

"These residents indicate the importance of being able to trust staff as a fundamental and necessary component of strong relationships. Their reflections suggest that therapeutic approaches will not be effective without a foundation of trust." (Author quote; Brubaker & Cleary, 2023).

Young people reported that the worker’s reliability evoked a deeper level of trust. By remaining predictable, young people could relax, feel safe and separate the relationship from other, inconsistent, relationships they had experienced.

"Reliability in this sense implies predictability, and so when practitioners act in a reliable manner, a child's sense of security is enhanced, and removes the uncertainty that comes from changeable and emotionally inconsistent parenting and upbringing." (Author quote; Cross, 2020).

This created a secure base, with young people describing how the relationship created a sense of security and reassurance when exploring and operating in the world.

"It’s fear of the unknown. I didn’t know and so that was really scary and having someone there to just tell you ‘that this is what’s going to happen, you go here, you do this’, cause doing it by yourself would’ve been ten times worse." (Participant quote; Trivasse, 2017).

As the relationship developed and the secure foundation strengthened, young people described becoming more mature and independent, experiencing a sense of autonomy. They reflected that, although they knew the worker was there, they felt they did not always need to rely on them and were able to do more for themselves.

"I think probably the staff but because you always know it’s there but maybe you don’t always take it and use it but it’s always there and you know that you can turn to them." (Participant quote; Tarrant & Torn, 2021).

However, supportive of the newfound independence was the security of knowing that the worker was still there, maintaining boundaries and holding expectations of the young person, whilst also continuing to keep them in mind.

"It felt good. You know I had someone there to not just, I wouldn’t say babysit me, I would say keep an eye on me […] to put some enforcement there […] you walk out of there feeling ten times better […] it was a care. It was—they wanna help you so much." (Participant quote; Trivasse, 2017).

Some young people discussed realising that the worker had done what they could, and it was now time for them to take ownership and responsibility of their future, feeling secure enough within themselves to manage this.

"So, it’s more a sort of … support, you know, I’m really grateful for but, it’s not the only problems I had. It’s like it’s my drinking, I just got to cut down on my drinking and all the other stuff like the offending stuff will kind of stop with it. So that’s what I’m more working on really, is to stop drinking to have a reason, more reasons not to drink and to have a bit more of a life." (Participant quote; Phoenix & Kelly, 2013).

#### Sub-theme 2: Showing Genuine Care by Going Above and Beyond

Young people were sensitive to professionals’ approaches to their jobs, discussing times when they perceived their actions as going beyond the role, showing that it was more than a job to them and that they genuinely cared.

"Obviously I think it’s part of her job but I do also think there’s a level that they care about everyone as well or they wouldn’t really be doing this job would they? I think although it is part of what they need to do they also do it because they do genuinely care." (Participant quote; Tarrant & Torn, 2021).

"They wanna help you so much. They go out of their way […] it’s not just a job, they enjoy their job, they wanna help people." (Participant quote; Trivasse, 2017).

This was reflected in the worker showing concern for the young person, that they weren’t asking or helping because it was part of the job, but that they wanted to make sure the young person was ok.

"Sam used to ask questions and be generally concerned and you could tell, you can tell when someone wants to know something and when they’re just asking it because they have to. I mean, Sam was dead concerned about me and wanted to help me basically." (Participant quote; Phoenix & Kelly, 2013)

Staff considering the young person’s wellbeing reflected that they mattered, which added to the feeling of being cared for.

"Though these descriptions of care may seem simplistic, the pervasiveness of residents’ insights regarding the power of their positive impact is important to note. Staff can make residents feel that they are cared for by showing them that they, and their wellbeing, matter."

(Author quote; Brubaker & Cleary, 2023).

Young people also discussed feeling that the worker cared when they held them mind and thought about them outside of scheduled session time.

"You can tell when someone cares because, like, they ring you up, like, before your interview, like say you had a job interview, they’d ring you up and wish you good luck. Stupid things like that." (Participant quote; Phoenix & Kelly, 2013).

By going above and beyond, the worker transformed and strengthened the relationship, with the young person holding them in a higher regard, just as they had for them.

"Going the extra mile meant that YOT practitioners were viewed as more than simply service providers." (Author quote; Trivasse, 2017)

## Discussion

The systematic review of seven qualitative studies thematically synthesised the experience and views of 150 young people in relation to the relationships they had with professionals in youth justice services. This study is, to the authors’ knowledge, the first to synthesise young peoples’ experience of professional relationships within youth justice services and identify what is important to their engagement. The findings are discussed in the context of the research questions (i) what are young people’s views and experiences of the relationships they have with youth justice professionals? and (ii) what factors have been identified by young people as important to their engagement with professionals?

Through the meta-synthesis of the data, four key themes were identified; Feeling valued and finding worth in the system; The reciprocal nature of understanding and respect; Nobody trying to help and no one to try for; and The importance of having one good person. Young people spoke about their experience and views of relationships with youth justice professionals and identified both positive and negative experiences. Some young people were also able to identify what was important to their engagement with these professionals.

For some young people, these relationships led to feeling a sense of worth in the system, where they felt seen, understood, respected and valued. The development of the adolescent’s value system not only builds identity but also supports the development of their self-esteem (Erikson, 1968; Sadock & Kaplan, 2009). Mirrored in our findings, young people not only discussed an appreciation for the care they received, but also reflected an ability to internalise it, holding value in themselves and improving their self-esteem. In comparison, for others, they experienced relationships which led to them experiencing a sense of inadequacy, due to feeling disregarded, unimportant and in some ways just a number in the system. Justice-involved youth can be “distrustful” of authority figures, because of previous experiences within the system where they felt disempowered (Burns & Creaney, 2023). Our study suggests that young people held preconceived judgements on how they would be treated by services and professionals, with primarily negative connotations in relation to this. Some young people even discussed surprise when they were treated kindly. This may indicate that young people enter services expecting unfair treatment, thus suggesting the potential for their defences to already be up, and therefore emphasising the importance of treating them with dignity and respect in order to foster a safe and supportive environment. The youth justice system has been described as a “traumatised and traumatising system”, with professionals’ reporting the burden of working with young people whose needs are not met, along with their projections of anxiety and risk (McElvaney & Tatlow-Golden, 2016).

Young girls involved in justice services rated respect as highly important, idealising someone who would listen to them and provide unconditional love and care (Belknap et al., 1997), whilst young men have been reported to value their worker’s honesty and sincerity and associated this with respect (Nugent, 2015). The participation of a young person is supported by mutual respect, because of its role in reducing “passive compliance” and promoting engagement, leading to more meaningful participation for young people (Duke & Fripp, 2022). Not only did our findings identify the importance of mutual respect, but we also found a reciprocation of care, whereby the young person felt morally obligated to requite the consideration of the professional, demonstrating the young person’s investment in the relationship. Our findings suggest that young people’s ideologies in relation to respect are complex, with young people not only recognising disrespect in how they were treated but also in the nuances of professional’s behaviours and reactions, for example, feeling as if they were being judged, labelled, and generalised based on their criminal history or behaviours. Unconditional positive regard is the process in which a therapist experiences a “warm acceptance” of the client’s experience and this being part of who they are, promoting acceptance of the self (Rogers, 2007). Professionals should endeavour to acknowledge young people as individuals, separate from their pasts, and avoid labels and categories that may further isolate or stigmatise them. Young people are aware of the inadequacies that they receive in interventions and feel unable to make changes (Duke & Fripp, 2022). This was apparent in our findings, with an atmosphere of lost hope echoed across aspects of the data. This is an important point in terms of the power of instilling hope in young peoples' lives. When young people feel like they can turn to their worker and hold hope that they will always be there to guide them through difficulty, they not only become able to rely on them, but they are inspired to do better and, in some cases, follow in their footsteps (Creaney, 2020).

Young people in youth justice services recognised the parental-like role that youth officers have played in their lives, how this has been helpful to them, and the appreciation that they have for this (Dako-gyeke et al., 2022). A key theme within our findings related to the secure base professionals provided young people, supportive of their exploration and development. A sense of security was facilitated through the professionals becoming ‘one good person’ in the young people’s lives, always being there in a non-judgemental and accepting capacity. Previous research has found that staff who were extremely consistent in enforcing rules, created an unsafe environment for young people in youth justice institutions, with rigidity to rules considered provoking and representative of staff inexperience (van der Laan & Eichelsheim, 2013). However, within our findings, young people discussed that boundaries enforced by staff, along with having someone to answer to or hold them responsible, was containing, further strengthening the secure foundations the relationship provided.

Findings from our study suggest that young people can differentiate between the professionals who are simply there to do their job and get paid, and those who communicate that their profession is more than just a job. Young people value professionals who are available when they most need it, at times when they have felt unsafe, or when they believed they may engage in criminal activity or use substances (Moore et al., 2013). Our findings reflected similar important factors in relation to engagement, including professionals showing genuine care for young people and providing an open and non-judgemental environment. We found that young people also valued being held in mind, small things like a professional checking in on them or remembering an important event in their lives, meant a lot to the young people, whilst youth workers showing genuine concern for their wellbeing made them feel that they mattered.

Persistency and ongoing engagement of the worker is paramount to the success of probation programmes and young people’s transition to adulthood (Nugent, 2015). Although our findings relate to the investment of the professionals and the genuineness they bring to their roles, it raises questions in relation to healthy workplace boundaries and the expectations placed on professionals to be able to maintain these relationships within the constraints of service hours and resources. This raises further questions on how professionals are supported by services in terms of supervision, reflective practice, and tailored training. Young people within the youth justice system are at greater risk of being traumatised or experiencing trauma. Without awareness of the growing impact of working with and directly supporting those affected by trauma, there is a risk that young people will be further traumatised, regardless of professional’s initiative or commitment (McElvaney & Tatlow-Golden, 2016).

Larkins and Wainwright (2020) found that relationships with youth justice workers facilitated the engagement of children and their ability to reflect and make changes. Our findings identified the role relationships have on reflection and change, as discussed by the young people. Our findings suggest that the secure base created by the relationship was supportive to young people when exploring their world. This aligns with attachment theories and may indicate the role of professional relationship in attachment change. There are three major functions of attachment relationships; to promote proximity seeking, provide a safe haven, and offer a secure base (Bowlby, 1958). Investigations into the prevalence of attachment disorders in a youth justice population found that 86% of the sample had experienced maltreatment, with the rates of actual or borderline attachment disorder being 52% (Moran et al., 2017). Attachment change requires deliberate effort in which participants have to experience identity transformation and changes in worth. For some, this may be facilitated by the relationship they have with their therapist (Dansby-Olufowote et al., 2020). Adolescence involves the process of moving away from relying on parental figures and the increasing importance of the social network in developing their identity (Blos, 1962). Our findings suggest that professionals relationships facilitated the development of trust and openness for the young people, which may support their ability to trust others, thereby potentially supporting the advancement of their social arena and their ability to become more independent.

### Limitations

Despite the sparsity of studies in the research area, the under-representation of the population, the specificity of the inclusion criteria, and notwithstanding the small number of studies included in the meta-synthesis, the voices of 150 young people were still represented within this review. The authors also acknowledge some limitations of the review. The included studies were divergent of one another in that they had different aims, research questions, qualitative methodologies and data collection methods. Not all the data available were collected for the primary purpose of investigating young people’s experience of relationships in youth justice services, therefore by changing the context of the data, the author may have altered the intended meaning and therefore there is a risk of bias in the findings. The potential for selective reporting in the studies used for this synthesis should also be considered. Whilst four of the included studies scored highly on quality assessment, one study that contributed to all themes scored poorly on the quality assessment. However, the authors note the subjectivity of quality assessments (Long et al., 2020). The studies were similar in that they were all from western countries and published in English. The review did not have capacity to translate and include non-English language data and therefore there may be cultural differences not represented in this review. Most studies were also from the UK and therefore may reflect a particular approach to youth justice that may not be indicative of experiences elsewhere geographically. The rationale for including young people specifically involved in statutory services, was to capture their experience of being involved in a relationship that was not of their initial designation. We focussed specifically on young people’s current experiences, however future research investigating different chronological viewpoints may be beneficial in comparing and contrasting young people’s experiences over time. To mitigate the risk of bias, blind screening was completed by two researchers on 100% of title and abstracts, with reviewer 1 screening 100% of full-text journals and reviewer 2 blind screening 20% of full-text journals. Rigorous reflexive coding and analysis was implemented, with all researchers contributing to this.

### Implications for Research and Practice

This research provides important insights directly from the young people involved in youth justice services. Policy makers, programme administrators and practitioners can learn some valuable lessons from the young people involved. Notwithstanding the relatively small evidence base to date, the findings suggest that there is potential for a system-wide commitment to relational-based practice, supported by training, supervision, and reflective practice opportunities for staff and organisations engaging with justice-involved youth. Programme development within these services too should be guided by a relational approach. Practitioners should create a non-judgemental environment where young people feel respected and treated equally and provide opportunities to develop a mutual understanding and common ground with the young people they work with. Creating a secure base built on trust and genuine care has also been found to be supportive to young people. Services should support staff in their roles, providing opportunities for supervision, training and reflective practice to promote a healthy dynamic within the working environment and job satisfaction. A framework for relational practice in Ireland has been developed by Fullerton et al. (2021), reflecting similar points and suggestions for service provisions and reform within the youth justice system in Ireland. In addition, evidence from an action research project undertaken within sixteen Youth Diversion Project case study sites in Ireland over a period of 3 years, has resulted in a new practice model and guidance for relationship building in youth justice (O’Meara Daly et al., 2025). This model and guidance highlight similar approaches that focus on building trust, providing safety, and person-centred care, to support youth justice practitioners building relationships that can help divert young people from crime. The importance of relational working has been identified and emphasised in this review, through the experiences of young people involved in youth justice services. However, there is still a relatively small evidence base in this area, regardless of the parallels in the literature on youth studies, social work and education. Future research aimed at exploring the impact of relational working in youth justice services, particularly capturing the voices of the young people, will be beneficial in building the evidence base and furthering our understanding of what works for young people in youth justice services.

## Conclusion

Through the meta-synthesis of the qualitative data exploring young people’s experiences of relationships with professionals within youth justice services, this review identified four key themes. The relationships young people have with professionals can support young people in finding value and worth in the system. For young people, factors that supported engagement included being valued and treated as a person, the reciprocation of understanding and respect and being shown genuine care from professionals. Young people reported positive experiences and outcomes when they felt they had ‘one good person’ in their lives, who created a secure base, supportive of their exploration and development.
